# Supplementary material for: Bacteria Colonies Modify Their Shear and Compressive Mechanical Properties in Response to Different Growth Substrates
Source: ACS Appl Bio Mater. 2024 Jan 9;7(12):7809–17. doi: 10.1021/acsabm.3c00907 (PMC11653398; doi:10.1021/acsabm.3c00907)
Supplement: Supplementary file 1 — mt3c00907_si_001.pdf [file mt3c00907_si_001.pdf]

Supporting Information

Bacteria colonies modify their shear and compressive mechanical properties in response to different growth substrates

Jakub A. Kochanowski[1], Bobby Carroll[1], Merrill E. Asp[1], Emma C. Kaputa[1], Alison E. Patteson[1*]

1- Physics Department and BioInspired Institute, Syracuse University, Syracuse, New York 13210, United States
* - Corresponding author. Email: aepattes@syr.edu

**Agar %**	**Agar G' Mean**	**Agar G' std error**	**Agar G" Mean**	**Agar G" std error**
1	1787.3	79.7	782.73	145.7
1.5	3375.5	198.5	3108.5	359.5
2	4512.5	383.52	2746.25	569.2

Table S1. Mechanical properties of 1, 1.5 and 2% agar.

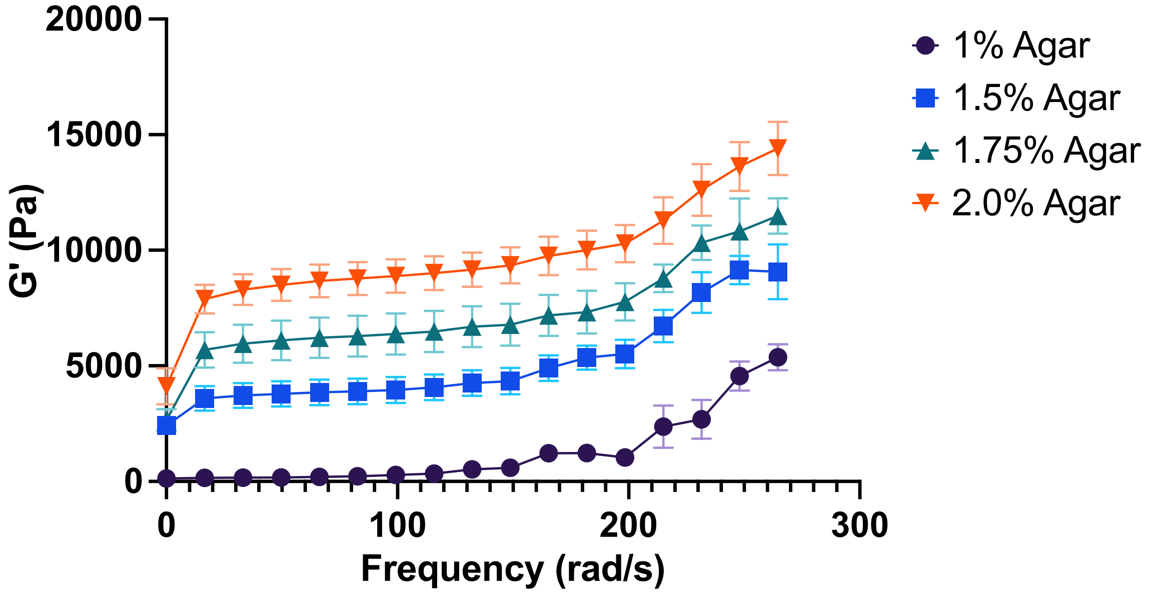

Fig S1. Frequency sweep for colonies grown on 1, 1.5, 1.75, and 2% agar.

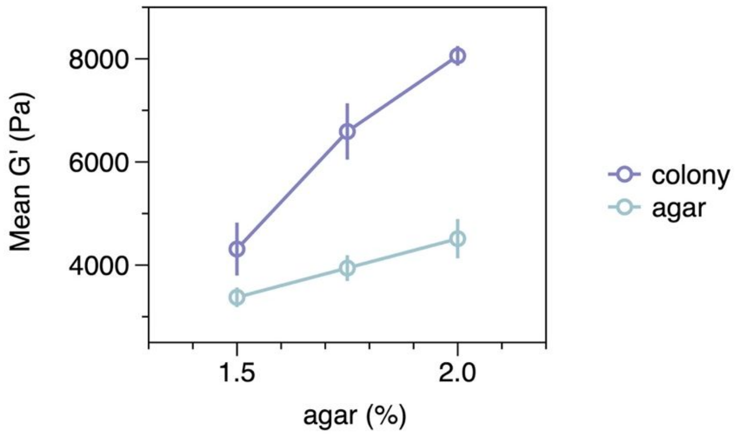

Fig S2. Mechanical properties of bacteria colonies compared to agar hydrogels. Bacteria hydrogels are stiffer than agar concentration and the difference grows with increasing agar concentration.

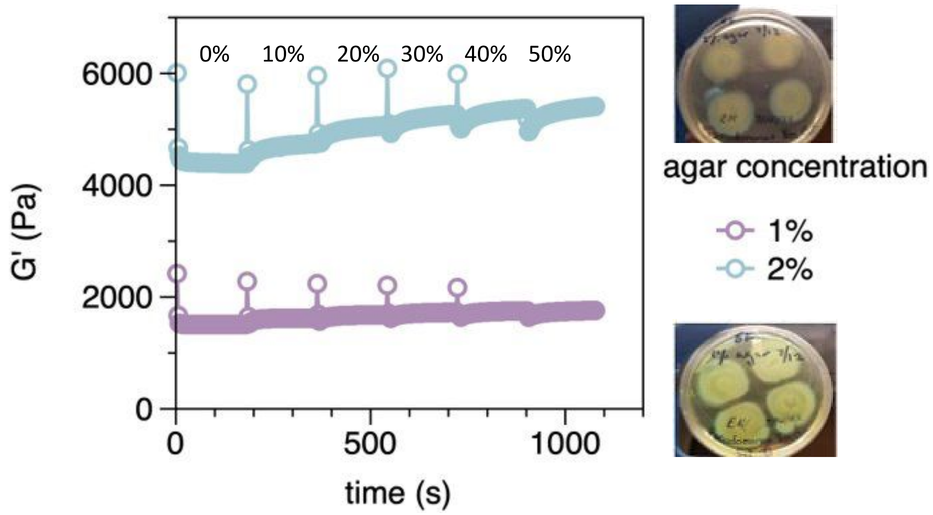

Fig S3. Representative time-series sequence data of *Pseudomonas aeruginosa* under uniaxial compression. Every 3 minutes the sample is compressed by 10% axial strain. Colonies grown on 1% agar do not show significant stiffening upon compression where colonies grown on 2% agar do.
